# A Comparative Study and Optimization of Camera-Based BEV Segmentation for Real-Time Autonomous Driving

**DOI:** 10.3390/s25072300

**Published:** 2025-04-04

**Authors:** Woomin Jun, Sungjin Lee

**Affiliations:** 1 Korea Electronics Technology Institute, Seongnam 13488, Republic of Korea; aplus912@keti.re.kr; 2 Department of Smart Automotive, Soonchunhyang University, Asan 31538, Republic of Korea

**Keywords:** autonomous driving, bird’s eye view, segmentation

## Abstract

This study addresses the optimization of a camera-based bird’s eye view (BEV) segmentation technique that operates in real-time within an embedded system environment while maintaining high accuracy despite limited computational resources. Specifically, it examines three technical approaches for BEV segmentation in autonomous driving: depth-based methods, MLP-based methods, and transformer-based methods, focusing on key techniques such as lift–splat–shoot, HDMapNet, and BEVFormer. A mathematical analysis of these methods is conducted, followed by a comparative performance evaluation using the nuScenes dataset. The optimization process was carried out in three stages: accuracy improvement, latency reduction, and model size optimization. In the first stage of the process, the three modules for BEV segmentation (encoder, view transformation, and decoder) were selected with the goal of maximizing mIoU performance. In the second stage, environmental variable optimization was performed through input resolution adaptation and data augmentation to improve accuracy. Finally, in the third stage, model compression was applied to minimize model size and latency for efficient deployment on embedded systems. Experimental results from the first stage show that the lift–splat–shoot view transformation model, based on the InternImage-B encoder and EfficientNet-B0 decoder, achieved the highest performance with 54.9 mIoU at an input image size of 448×800. Notably, the lift–splat–shoot view transformation model with the InternImage-T encoder and EfficientNet-B0 decoder demonstrated performance of 53.1 mIoU while achieving high efficiency (51.7 ms and 159.5 MB, respectively). The application of the second stage revealed that increasing the input resolution does not always lead to improved accuracy, and there is an optimal resolution size depending on the model. In this study, the best performance was achieved with an input image size of 448×800. During the third stage, FP16 quantization enabled a 50% reduction in memory size and decreased latency while maintaining similar or identical mIoU performance. When deployed on the NVIDIA AGX Orin device, which operates under power constraints, energy efficiency improved, although it resulted in higher latency under certain power supply conditions. As a result, the InternImage encoder-based lift–splat–shoot technique was shown to achieve the highest accuracy performance relative to latency and model size. This approach outperformed the original method by achieving a 29.2% higher mIoU while maintaining similar latency performance and reducing memory size by 32.2%.

## 1. Introduction

With the advancements in artificial intelligence technologies, autonomous driving and robotics are rapidly progressing towards commercialization [1,2]. Initially, the development of artificial intelligence in this field began with image recognition technologies, focusing on image classification, object recognition, and object segmentation for initial situation awareness in autonomous driving. However, the trend in technology is now evolving into a comprehensive situational awareness system, extending to include reinforcement learning, transformer-based time series processing, and multi-modal learning as foundational elements for sensor fusion-based situational awareness, path planning, and end-to-end learning [1,2]. In particular, the ability to reinterpret and transform 2D input images from a 3D situational awareness and BEV perspective is becoming increasingly important for facilitating practical autonomous driving decisions rather than relying solely on 2D object detection and segmentation techniques. Associated with this, improvements in accuracy metrics and real-time processing capabilities in embedded systems are also gaining significance [3,4,5]. Lightweight deep learning techniques based on real-time embedded systems also play a crucial role in the realization of autonomous driving technology. Technologies such as light convolution modules, factorization, quantization, pruning, and knowledge distillation are extensively researched and are vitally important for practical implementation within the field of autonomous driving. These techniques contribute significantly to reducing the computational load and enhancing efficiency, making them indispensable for deploying sophisticated autonomous driving functions in real-world applications [1,2].

This study focuses on optimizing the accuracy of BEV transformation and segmentation technologies, which are crucial for practical autonomous driving, as well as lightweight real-time deep learning optimization techniques. To analyze the stability and efficiency of these technologies, the accuracy of BEV segmentation, processing time, and model size were measured. As part of the optimization strategy, various candidate technologies were explored for each key module, including the image encoder, view transformation, and BEV decoder, to identify the optimal combination that maximizes accuracy. Additionally, FP16 quantization was applied to maximize efficiency, enabling real-time operation within the resource-constrained environment of embedded systems for autonomous driving.

The main contributions of this paper can be summarized as the following three points:

- *Selection of the BEV image encoder, view transformation, and BEV decoder models:* Based on the evaluation of mIoU accuracy, model size, and latency performance, the optimal image encoder, view transformation, and BEV decoder models were determined.
- *Determination of the BEV input image size and data augmentation:* The optimal image size was selected through a performance comparison across resolutions of 224×480, 448×800, and 672×1200. Additionally, suitable data augmentation strategies were identified.
- *Lightweight model optimization:* Quantization techniques were applied to the model, and its accuracy and latency were measured to implement a lightweight optimization strategy for real-time autonomous driving embedded systems. Furthermore, the BEV segmentation performance was analyzed on-device, specifically deploying the model on the NVIDIA AGX Orin platform to assess its real-world applicability and to derive corresponding deployment strategies.

## 2. Related Work

In autonomous driving, 2D BEV transformation technology for the surrounding 3D environment has primarily been implemented using inverse perspective mapping (IPM) [5]. This approach involves converting 2D perspective view (PV) input images into 3D spatial coordinates using the camera’s intrinsic and extrinsic parameters, followed by a projection onto a 2D plane to generate BEV images. However, various environmental factors that affect camera capture, such as sunlight and reflections, weather conditions, dynamic objects, and occlusions, can limit the accuracy of this projection. Additionally, since IPM operates under the assumption that all points lie on the ground, it can introduce errors in height estimation. To address the inherent limitations of IPM-based BEV transformation, several studies have emerged that apply deep learning techniques to improve performance.

BEV transformation techniques can be categorized based on how they convert 2D PV images from each camera into 3D spatial coordinates. These methods include depth-based, MLP-based, and transformer-based approaches.

Depth-based methods [6,7,8,9] transform 2D images into voxel or point-cloud representations of 3D data through depth estimation, which are then converted to BEV. Representative methods include the orthographic feature transform (OFT) [6], Lift–splat–shoot (LSS) [7], pseudo-LiDAR [8], and simple BEV [10]. OFT [6] voxelizes feature information extracted from 2D PV images using a ResNet18 encoder and transforms them into BEV. Pseudo-LiDAR [8] generates a depth map from 2D images through depth estimation, converting each pixel into a 3D point cloud.

Lift–splat–shoot (LSS) [7] tackles BEV transformation for end-to-end learning, with the transformation occurring in the lift and splat stages. The lift stage converts 2D PV images into a 3D frustum, obtained via outer product calculations between depth probability distributions and image encoder outputs. The splat stage then transforms this 3D frustum into BEV’s pillar-based features using the PointPillars [11] technique, which generates a tensor in the C×H×W format suitable for standard CNN processing, followed by segmentation. The LSS method has inspired further variations, including simple BEV [10], which applies bilinear sampling at each pixel’s projection location to form 3D voxels during the lift stage.

Meanwhile, MLP-based and transformer-based methods have emerged, applying neural network architectures for view transformation to learn the mapping relationship between 2D PV images and 3D feature space.

MLP-based methods learn the relationship between feature locations in 2D PV and BEV perspectives using MLPs [12,13,14,15]. VPN [13] employs a two-layer MLP to transform surrounding image feature maps into BEV feature maps, followed by multi-view fusion from other cameras. HDMapNet [14] fuses image feature maps from cameras and point cloud feature maps from LiDAR to generate BEV feature maps through a two-layer MLP. HDMapNet can also be implemented using only cameras, without LiDAR, for MLP-based view transformation and BEV feature map generation.

Transformer-based methods use attention mechanisms to learn the mapping relationship between PV and BEV coordinates, performing the transformation [16,17,18,19]. While similar to MLP-based methods in the sense that they convert PV to BEV without relying on camera models, transformer-based approaches differ in that their weight matrices are data-dependent and positional encoding is applied during input, making them sequence-independent and utilizing a query-based attention mechanism [12]. DETR3D [16] performs direct 3D recognition without depth estimation, linking 3D object queries to 2D images through camera transformation matrices to generate BEV feature maps. PolarFormer [17] applied polar coordinate transformation, which is intuitive for 3D recognition, to improve performance, utilizing cross-attention mechanisms to integrate multiple image feature maps into BEV feature maps.

BEVFormer [18] applied attention mechanisms across both spatial and temporal domains. In the spatial domain, a deformable attention-based spatial cross-attention (SCA) model was developed to ensure each BEV query searches only within regions of interest, improving resource efficiency. In the temporal domain, a temporal self-attention (TSA) model was developed to associate current BEV queries ($B_{t}$) with historical BEV features ($B_{t-1}$), enabling temporal fusion.

Thus, this study focuses on optimizing BEV performance and applying lightweight deep learning techniques in real-time for three key BEV transformation approaches: (1) depth-based methods (e.g., LSS), (2) MLP-based methods (e.g., HDMapNet), and (3) transformer-based methods (e.g., BEVFormer). To achieve this, various candidate techniques are explored for each module of the BEV transformation and segmentation pipeline, including the image encoder, view transformation, and BEV decoder, to determine the optimal combination for achieving the highest accuracy.

## 3. System Model

**Figure 1:**
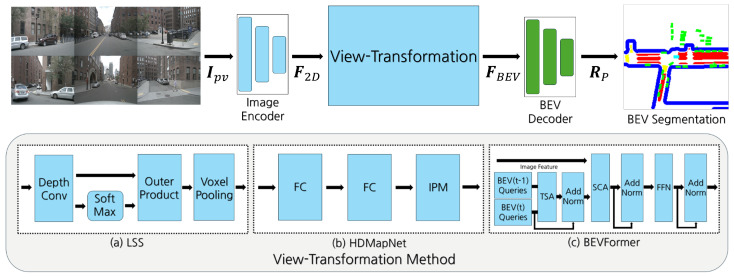
Overall operation modules in BEV segmentation for autonomous driving. The figure illustrates the overall operation module architecture from multi-camera images to BEV (bird’s eye view) segmentation across different models: (**a**) LSS, (**b**) HDMapNet, and (**c**) BEVFormer. Each model employs a distinct set of modules to process image features and transform them into BEV representations, enabling effective scene understanding for autonomous driving applications.

The model architecture used in this paper includes LSS, HDMapNet, and BEVFormer. Figure 1 illustrates the structure of each system. The BEV transformation model used in this paper is based on a three-module architecture consisting of an image encoder, view transformation, and BEV decoder. The structure of each module is analyzed in Section 3.1, Section 3.2 and Section 3.3.

### 3.1. Image Encoder

This module extracts features from the PV images obtained from one or more cameras using an image classification backbone. The image encoder is a common module for BEV segmentation and is uniformly applied across the LSS, HDMapNet, and BEVFormer models.

The mathematical model for the image encoder is provided in Equation (1).

(1) $$\begin{array}{c} F_{2D}\left(\hat{u},\hat{v}\right)=E\left(I_{PV}\left(u,v\right)\right), \end{array}$$

where $I_{PV}$ represents the PV images from multiple cameras, *u* and *v* are the coordinates of the PV images, E(·) is the image encoder, F2D represents the features extracted by the image encoder, and $\hat{u}$ and $\hat{v}$ are the feature coordinates from the encoder.

The image encoder E(·) is modeled using EfficientNet [20], transformer [21], and InternImage [22], as represented in Equation (2),

(2) $$\begin{array}{c} E(\cdot )\in \{\mathrm{EFN},\mathrm{MiT},\mathrm{ITN}\}, \end{array}$$

where EFN is the EfficientNet model [20], MiT is the transformer-based backbone used in [21], and ITN is the transformer-based backbone known as InternImage [22].

### 3.2. View Transformation

The view transformation module converts the PV features extracted by one or more image encoders into BEV features. The input to the view transformation is the 2D PV image features, and the output is a BEV feature of size C×H×W.

As mentioned earlier, view transformation methods can be categorized into depth-based methods, MLP-based methods, and transformer-based methods. In this paper, we analyze and evaluate the performance of representative approaches, including LSS, HDMapNet, and BEVFormer, through mathematical analysis and experimentation.

#### 3.2.1. Depth-Based Method

In LSS, a 3D frustum is generated through the outer product of the PV feature values and the latent depth distribution, which is then transformed into BEV using the voxel pooling (VP) method from PointPillars. In this process, the feature map is passed through Depth-Conv and then through a Softmax layer, with the resulting matrix used to add a depth dimension through an outer product with the unprocessed matrix. This additional depth dimension is treated as voxel data and transformed into BEV features via PointPillars’ VP method, following the structure of OFT.

The mathematical model for generating a 3D frustum via the outer product of the PV features and latent depth distribution is shown in Equation (3).

(3) $$\begin{array}{c} F_{3D}\left(x,y,z\right)={\left[F_{2D}\left(\hat{u},\hat{v}\right)⊗P_{D}\left(\hat{u},\hat{v}\right)\right]}_{x,y,z}, \end{array}$$

where $P_{D}$ represents the depth distribution.

Next, VP is applied on the BEV plane, following the principles of the PointPillars method. Specifically, each point is assigned to the nearest pillar, and sum pooling is performed to generate a C×H×W tensor, which is processed by a standard CNN for BEV inference.

(4) $$\begin{array}{c} F_{BEV}\left(x,y\right)=\mathrm{VP}\left(F_{3D}\left(x,y,z\right)\right), \end{array}$$

where VP(·) is the voxel pooling operation, and point (x,y) exists on the BEV plane with dimensions W×H.

#### 3.2.2. MLP-Based Method

In methods like HDMapNet, PV image features $F_{2D}\left(\hat{u},\hat{v}\right)$ are first transformed into the camera coordinate system (CCS) and then into BEV.

The transformation from PV to CCS is represented by Equation (5).

(5) $$\begin{array}{c} F_{3D}\left(x,y,z\right)=T_{MLP}\left(F_{2D}\left(\hat{u},\hat{v}\right)\right), \end{array}$$

where $T_{MLP}$ is the multi-layer perceptron which models the relation of any two pixels between PV and CCS.

Then, the transformation from CCS to top-down is performed by geometric projection with camera extrinsics:

(6) $$\begin{array}{c} F_{BEV}\left(x,y\right)=T_{PRO}\left(F_{3D}\left(x,y,z\right),\left[R,T\right],K\right), \end{array}$$

where $T_{PRO}$ is the geometric projection function with camera extrinsic parameters. *R* and *T* are extrinsic parameters, and *K* is the intrinsic parameter. The extrinsic and intrinsic parameters define the mapping from coordinates (x,y) to local pixel coordinates (u,v).

#### 3.2.3. Transformer-Based Method

BEVFormer takes both the spatial and temporal relationships in BEV into account through deformable attention. Temporal relationships are derived via temporal cross-attention between images, while spatial cross-attention is used to extract spatial relationships within a single image [18].

The view transformation module of BEVFormer utilizes the output of the image encoder $F_{2D}$, the history of BEV features $F_{BEV,t-1}$, and BEV queries $Q(F_{BEV,t})$. Here, BEV queries $Q(F_{BEV,t})$ inquire about the spatial information from the multi-camera features $F_{2D}$ via spatial cross-attention.

In addition, $Q(F_{BEV,t}^{p})$, located at p=(x,y) on the BEV plane, is responsible for the corresponding grid cell region. By default, the center of BEV features corresponds to the position of the ego vehicle.

First, BEVFormer lifts each query from the BEV plane into a pillar-like query, samples $N_{ref}$ 3D reference points from the pillar, and projects these points onto the 2D image.

First, temporal self-attention (TSA) is performed as follows:

$$\mathrm{TSA}\left(Q\left(F_{BEV,t}^{p}\right),\left\{Q\left(F_{BEV,t}\right),F_{BEV,t-1}^{\prime }\right\}\right)=\sum_{V\in \{Q\left(F_{BEV,t}\right),F_{BEV,t-1}^{\prime }\}}\mathrm{DefAtt}\left(Q\left(F_{BEV,t}^{p}\right),p,V\right),$$

where $F_{BEV,t-1}^{\prime }$ is the aligned BEV history to $Q(F_{BEV,t})$ based on ego-motion to ensure that the features at the same grid correspond to the same real-world location, i.e., $F_{BEV,t-1}^{\prime }=F_{BEV,t-1}-P_{REF}$, where $P_{REF}$ is the origin point corresponding to the same real-world location.

Additionally, spatial cross-attention (SCA) is performed as follows:

$$\mathrm{SCA}\left(Q\left(F_{BEV,t}^{p}\right),F_{BEV,t}\right)=\frac{1}{|V_{\mathrm{hit}}|}\sum_{i\in V_{\mathrm{hit}}}\sum_{j=1}^{N_{\mathrm{ref}}}\mathrm{DefAtt}\left(Q\left(F_{BEV,t}^{p}\right),P\left(p,i,j\right),F_{BEV,t}^{i}\right),$$

where *i* indexes the camera view, *j* indexes the reference points, and $F_{BEV,t}^{i}$ represents the features from the *i*-th camera view. For each BEV query $Q(F_{BEV,t}^{p})$, BEVFormer uses the projection function P(p,i,j) to obtain the *j*-th reference point on the *i*-th view image.

The projection function P(p,i,j), which performs the projection of the *j*-th 3D point to the *i*-th view, is defined as follows:

(7) $$\begin{array}{cc} \; & P\left(p,i,j\right)=\left(x_{ij},y_{ij}\right),\\ \mathrm{where}\; & z_{ij}\cdot {\left[\begin{array}{ccc} x_{ij} & y_{ij} & 1 \end{array}\right]}^{T}=T_{i}\cdot {\left[\begin{array}{cccc} x^{\prime } & y^{\prime } & z_{j}^{\prime } & 1 \end{array}\right]}^{T}. \end{array}$$

Using the above TSA and SCA modules, the view transformation module of BEVFormer is structured as shown in Figure 1c.

The view transformation module output for LSS is as follows:

(8) $$F_{BEV}^{LSS}=\mathrm{VP}\circ \left(P_{D}⊗F_{2D}\right).$$

The output of the view transformation module for HDMapNet is as follows:

(9) $$F_{BEV}^{HMN}=T_{PRO}\circ T_{MLP}\circ F_{2D}.$$

The output of BEVFormer’s view transformation module is as follows:

(10) $$\begin{array}{cc} F_{BEV}^{BFR}= & {\mathrm{AN}}_{s}(\mathrm{FFN}({\mathrm{AN}}_{s}(\mathrm{SCA}({\mathrm{AN}}_{c}(\\ & \;\;\;\;\;\;\;\;\;\mathrm{TSA}(Q\left(F_{BEV,t}^{p}\right),{\left\{Q\left(F_{BEV,t}\right),F_{BEV,t-1}\right\}}^{\prime }),\;\\ \; & \;\;Q\left(F_{BEV,t}\right)),\;F_{2D})))). \end{array}$$

Here, ${\mathrm{AN}}_{s}$ refers to the add and normalization operation applied to a single input, ${\mathrm{AN}}_{c}$ refers to the add and normalization operation applied to two inputs, and FFN represents the feed forward network operation.

### 3.3. BEV Decoder

The BEV decoder module is responsible for generating the final BEV image and segmentation output from the extracted BEV features. This BEV decoder module is common across LSS, HDMapNet, and BEVFormer models in BEV segmentation.

The mathematical model for the BEV decoder is as follows:

(11) $$\begin{array}{c} R_{P}=D\circ V\circ E\left(I_{PV}\right) \end{array}$$

$$\begin{array}{ccc} D(\cdot ) & \in & \{EFN,MiT,ITN\},\\ V(\cdot ) & \in & \{F_{BEV}^{LSS},F_{BEV}^{HMN},F_{BEV}^{BFR}\}, \end{array}$$

where D(·) refers to one of the candidate BEV decoder models, and V(·) refers to one of the candidate view transformation models.

## 4. Performance Enhancement Strategy

### 4.1. Evaluation Metrics

The performance evaluation metrics used for accuracy are IoU (intersection over union) and mIoU (mean intersection over union). IoU is measured as follows:

(12) $$\mathrm{IoU}\left(R_{P},R_{G}\right)=\frac{|R_{P}\cap R_{G}|}{|R_{P}\cup R_{G}|},$$

where $R_{P}$ represents the set of pixels predicted as the target class by the model, and $R_{G}$ represents the set of pixels actually belonging to the target class.

For the BEV segmentation task in this study, the target classes include lane, crosswalk, bound, and car. Thus, the above IoU formula is applied for these classes, and mIoU is used to comprehensively evaluate the accuracy across all classes:

(13) $$\mathrm{mIoU}=\frac{1}{N_{c}}\sum_{c=1}^{N_{c}}\frac{|R_{P,c}\cap R_{G,c}|}{|R_{P,c}\cup R_{G,c}|},$$

where $N_{c}$ is the total number of classes, i.e., lane, crosswalk, bound, and car. $R_{P,c}$ and $R_{G,c}$ represent the predicted and ground-truth sets for the *c*-th class.

Additionally, the efficiency of the BEV segmentation technique is measured in terms of latency (ms) and model size (MB).

### 4.2. BEV Enhancement Methodology

This section presents the strategy for improving BEV performance. The process consists of three stages: maximizing accuracy through optimal input resolution and data augmentation selection, as shown in Figure 1, followed by model compression based on these optimizations.

#### 4.2.1. Model Determination

Based on the BEV operation modules presented in Section 3.1, the IoU and mIoU for each class are computed to select the optimal image encoder, view transformation model, and BEV decoder model with the highest accuracy performance.

At this stage of model determination, data augmentation is not applied, and the input resolution is set to 448×800.

The goal of BEV segmentation is to maximize the mIoU between $R_{P}$ and $R_{G}$, as formulated in Equation (14).

(14) $$\max\{\mathrm{mIoU}\left(R_{P},R_{G}\right)\}.$$

To achieve this, Equation (14) can be reformulated as shown in Equation (15):

(15) $$\begin{array}{ccc} E^{*},V^{*},D^{*} & = & \arg\max_{E,V,D}\left\{\mathrm{mIoU}\left(D\circ V\circ E\left(I_{PV}\right),R_{G}\right)\right\},\\ \mathrm{subject}\;\mathrm{to}\; & & E\in \{\mathrm{EFN},\mathrm{MiT},\mathrm{ITN}\},\\ & & V\in \{F_{BEV}^{LSS},F_{BEV}^{HMN},F_{BEV}^{BFR}\},\\ & & D\in \{\mathrm{EFN},\mathrm{MiT},\mathrm{ITN}\}. \end{array}$$

#### 4.2.2. Model Enhancement

In this stage, the performance of mIoU is further enhanced by varying the input resolution and data augmentation techniques. Input resolutions of 224×480, 448×800, and 672×1200 are experimented with to determine the optimal input resolution for the given BEV segmentation model. For data augmentation, both filter-based and geometric-based methods are tested to identify the optimal augmentation technique.

#### 4.2.3. Model Compression

Finally, after achieving optimization in terms of IoU accuracy, model compression was applied by considering latency and model size. Several quantization techniques (e.g., baseline quantization, full integer quantization, FP16 quantization, quantization-aware training, and pruning quantization) were considered. However, based on the results from prior research [23], only FP16 quantization was selected as the optimal choice, considering the trade-offs between compression rate and accuracy degradation.

According to the results of the study [23], the performance of no quantization (NQ), baseline quantization (BLQ), full integer quantization (FIQ), FP16 quantization (F16), and quantization-aware training (QAT) can be summarized, as shown in Table 1. However, since accuracy, model size, and latency are all critical parameters in the autonomous driving mobility domain, FP16 quantization is considered the most suitable option, as it offers advantages over the baseline across all these aspects.

## 5. Simulation Result

The experiments were conducted using the nuScenes dataset [24], with image resolutions set to 224×480, 448×800, and 672×1200. The computational environment for the experiments utilized dual RTX 4090 GPUs for training and a single RTX 4090 GPU for inference, programmed using PyTorch 1.12.0. Cross-entropy loss and the AdamW optimizer were used for training, with learning rate adjustments made via the step LR method. The performance of each model was evaluated based on the highest mIoU achieved over 30 epochs.

For the analysis of BEV segmentation performance, the IoU of classes lane, crosswalk, bound, and car, as well as the average mIoU, were evaluated for various model structures, encoder structures, and decoder structures, as shown in Table 2 and Table 3. The BEV transformation and segmentation target area for the PV images from six multi-cameras was set to 100 m × 100 m. The compared model architectures included lift–splat–shoot (LSS), HDMapNet (HMN), and BEVFormer (BFR), while the evaluated encoders were EfficientNet (EFN) B0/B4, mix-transformer (MiT) B0/B2, and InternImage (ITN) T/B. The performance was measured using IoU and latency values. In addition, Table 4 presents the accuracy performance of the image encoder models used in this study on the ImageNet dataset.

**Figure 2:**
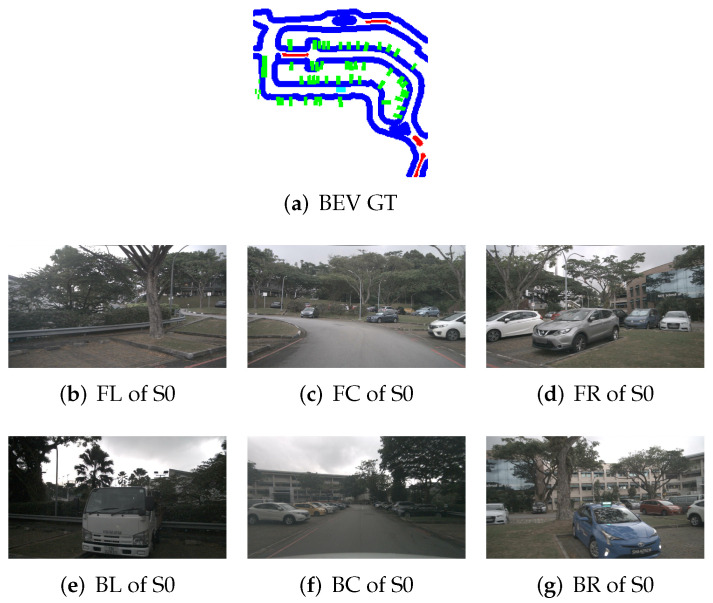
Surrounded view images of scene S0 and its corresponding BEV ground truth image from the NuScenes dataset (cyan: ego vehicle, red: lane, blue: boundary, and green: car).

**Figure 3:**
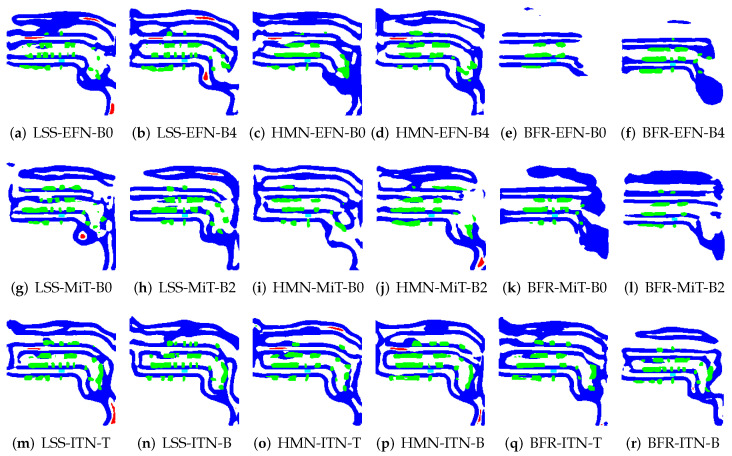
Example results of applying the BEV segmentation model to 224×480 image inputs in the NuScenes dataset (cyan: ego vehicle, red: lane, blue: boundary and green: car).

**Figure 4:**
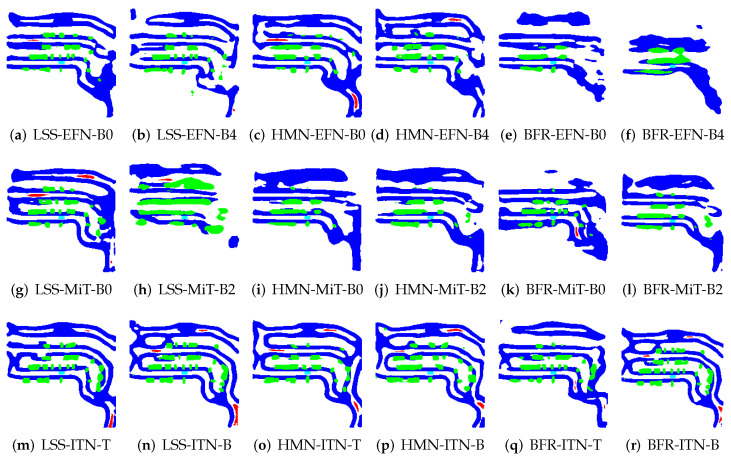
Example results of applying the BEV segmentation model to 448×800 image inputs in the NuScenes dataset (cyan: ego vehicle, red: lane, blue: boundary and green: car).

**Figure 5:**
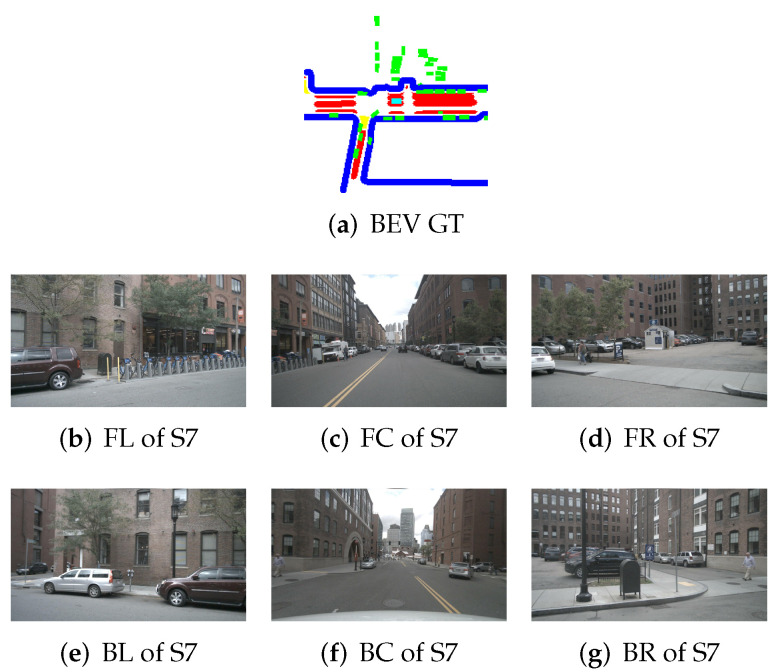
Surround view images of scene S7 and its corresponding BEV ground truth image from the NuScenes dataset (cyan: ego vehicle, red: lane, blue: boundary, yellow: crosswalk, and green: car).

**Figure 6:**
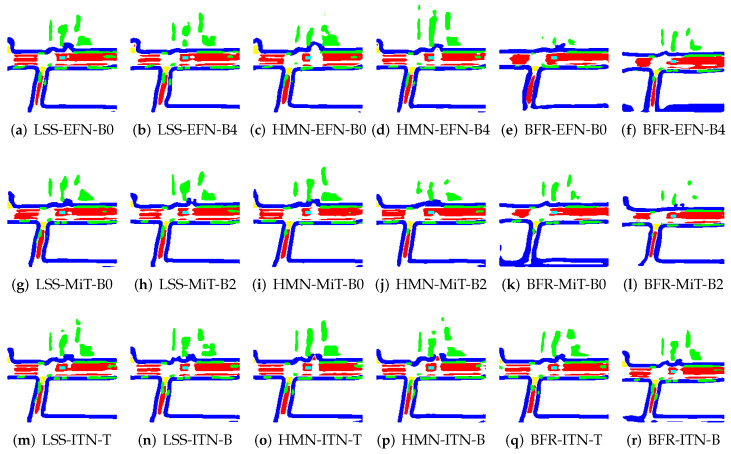
Example results of applying the BEV segmentation model to 224×480 image inputs in the NuScenes dataset (cyan: ego vehicle, red: lane, blue: boundary, yellow: crosswalk, and green: car).

**Figure 7:**
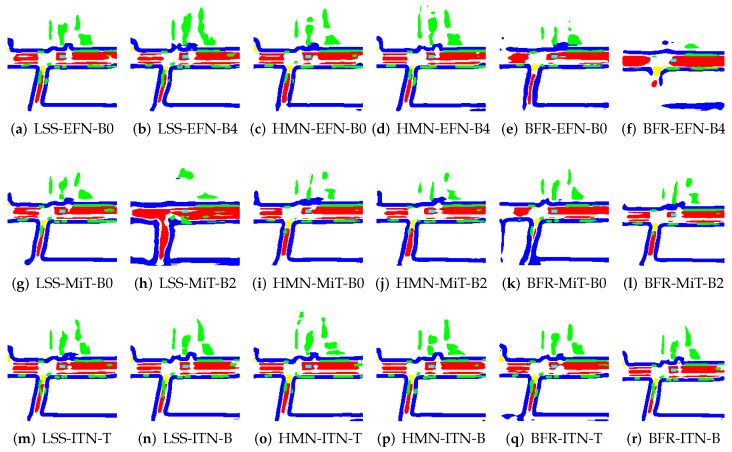
Example results of applying the BEV segmentation model to 448×800 image inputs in the NuScenes dataset (cyan: ego vehicle, red: lane, blue: boundary, yellow: crosswalk, and green: car).

To illustrate the application of BEV transformation and BEV segmentation, two distinct scenarios were established: a high-density traffic area and a crosswalk-distributed urban environment. First, the camera scene and ground truth (GT) image for a high vehicle-density area are shown in Figure 2. The corresponding BEV transformation and BEV segmentation results for five target classes are presented in Figure 3 and Figure 4. Additionally, the camera scene and GT image for a crosswalk-distributed urban area are shown in Figure 5. The BEV transformation and segmentation results for five target classes in this scenario are illustrated in Figure 6 and Figure 7. Figure 3 and Figure 4 correspond to the high-density traffic area, illustrating the outcomes of BEV transformation and segmentation for input image sizes of 224×480 and 448×800. Similarly, Figure 6 and Figure 7 correspond to the crosswalk-distributed urban area, demonstrating the impact of BEV transformation and segmentation under the same input image size conditions. The segmentation results are color-coded as follows: cyan—ego vehicle, red—lane, blue—boundary, yellow—crosswalk, green—other vehicles. To quantitatively evaluate the model’s performance, mIoU was computed as the mean IoU across all target classes, including lane, crosswalk, boundary, and car. Additionally, latency was measured in milliseconds (ms), while model size was expressed in megabytes (MB).

Model compression was evaluated using FP16 quantization [23], and on-device performance comparisons were made using the NVIDIA AGX Orin platform, with GPU power limits set to 30 W and 50 W [25].

### 5.1. Model Determination

Table 2 shows the mIoU, latency, and size performance of BEV segmentation models with different image encoders and input image sizes (224×480, 448×800). The BEV decoder used for these analyses was EfficientNet-B0.

As shown in the results, the ITN-B model achieved the highest mIoU accuracy across all test sets. However, it exhibited the highest latency and largest model size, indicating inefficiency relative to its performance. On the other hand, the ITN-T model demonstrated superior mIoU performance in terms of model size and latency compared to ITN-B. While ITN-B provided a slight improvement in mIoU (approximately 0~2), it required approximately three times the memory size and nearly double the latency. Specifically, in the LSS (448×800) setup, ITN-T achieved a high mIoU of 51.6 with a relatively small model size of 159.5 MB and an excellent latency of 51.7 ms. In contrast, ITN-B recorded a slightly higher mIoU of 53.4, but with a larger model size of 427.3 MB and a higher latency of 95.5 ms.

It is worth noting from Table 4 that ITN-T is an efficient model, offering high accuracy with a relatively small number of parameters. Similarly, EFN-B0 and EFN-B4 also demonstrate practical value, achieving stable accuracy performance while maintaining a remarkably low parameter count.

Similarly, for BFR (448×800), ITN-T demonstrated the best trade-off between accuracy and efficiency, with a model size of 179.14 MB, latency of 64.5 ms, and mIoU of 49.3. Compared to the performance of ITN-B (507.1 MB model size, 106.8 ms latency, and 50.7 mIoU), ITN-T provided significantly better efficiency, making it an optimal choice for real-time autonomous driving systems that are sensitive to model size and latency constraints. By providing similar mIoU performance while significantly reducing the model size compared to ITN-B, ITN-T’s advantages become more pronounced in resource-constrained systems or applications that demand high efficiency.

Additionally, EFN-B0 exhibited the smallest model size, which could be advantageous for systems with memory constraints. The MiT-B0 model demonstrated adequate performance across all test sets in terms of latency and memory size, positioning it as a well-balanced option suitable for real-time applications. However, despite the advantages of smaller model sizes and faster latency, models other than ITN-T/B—specifically EFN-B0, EFN-B4, MiT-B0, and MiT-B2—showed relatively lower accuracy, rendering them less reliable for use in autonomous driving scenarios.

Looking at class-specific IoU performance across encoder models, it is evident that static objects related to driving regions, such as bound and lane, generally exhibited higher IoU performance. However, despite being a static object, the crosswalk class showed lower performance due to its structural similarity to other static classes (e.g., bound and lane). Dynamic objects like cars had the lowest recognition performance. Among the image encoders, ITN-T/B models showed more balanced performance across all classes, with relatively better recognition of the crosswalk and car classes.

In particular, the crosswalk class exhibits lower accuracy, emerging as a bottleneck for the overall mIoU performance. This is due to the structural characteristics of crosswalks, which are similar to lanes but possess more complex features, leading to a performance dependency on image resolution. This can be observed in the crosswalk recognition results shown in Figure 6 and Figure 7. Specifically, while the crosswalk class is accurately recognized at a higher resolution (448×800), the IoU performance decreases at a lower resolution (224×480) due to the lack of texture information, especially for objects at greater distances where images are captured with lower resolution. Moreover, despite the lower resolution, the ITN-T/B models demonstrate stable performance levels comparable to those observed at higher resolutions. This suggests that, for accurate recognition of complex classes like crosswalk, it may be more effective to utilize encoder structures with larger, more sophisticated architectures, such as ITN, rather than simpler models like EFN, to ensure safer autonomous driving.

The ITN-B model demonstrated the highest performance in car detection, which is crucial for autonomous vehicles in detecting and tracking moving objects. The ITN-B model exhibited superior performance in high-resolution scenarios (448×800), indicating its advantage in handling complex traffic environments. This represents a significant improvement over the original LSS performance of 32.06 mIoU with ResNet, suggesting that optimizing the image encoder model alone can lead to an increase of approximately 6~7 mIoU.

Table 3 presents the mIoU, latency, and model size performance for various BEV decoders when using the LSS ITN-T image encoder with an input size of 224×480. As the results indicate, there is minimal variation in accuracy across different decoder models, but the model sizes show significant differences. In this study, EfficientNet-B0 was identified as the most suitable BEV decoder model for practical use, as it offers the smallest model size while maintaining the highest mIoU performance.

The LSS model demonstrated the best performance for BEV segmentation at both 224×480 and 448×800 resolutions. Specifically, at the lower resolution of 224×480, LSS outperformed other models, with HDMapNet and BEVFormer following closely with comparable performance. At the higher resolution of 448×800, LSS continued to exhibit superior performance. However, BEVFormer showed an improvement and achieved results nearly equivalent to those of LSS.

### 5.2. Model Enhancement

In this stage, additional performance improvements are derived based on the previously selected optimal encoder, VT model, and decoder, focusing on input image resolution and data augmentation.

First, we explore performance enhancements based on image input resolution. As previously mentioned, LSS demonstrates the best performance at both 224×480 and 448×800. Specifically, at 224×480, HDMapNet and BEVFormer exhibit comparable performance to each other, following LSS. At 448×800, BEVFormer performs slightly better than HDMapNet, with LSS still maintaining the highest performance. Table 5 presents the mIoU, latency, and model size performance for LSS, HDMapNet, and BEVFormer models with the ITN-T encoder at an input resolution of 672×1200. The results indicate that at 672×1200, LSS achieves 52.7 mIoU, and BEVFormer achieves 52.8 mIoU. These values are lower than those obtained with the 448×800 resolution using the InternImage encoder-based LSS, suggesting that increasing the input resolution does not necessarily lead to improved accuracy and that there is an optimal input size depending on the model and data characteristics. Additionally, while LSS performs best at lower input resolutions, BEVFormer outperforms LSS at higher resolutions. From a latency perspective, it is evident that as the input image size increases, latency increases for all models (LSS, HDMapNet, and BEVFormer). This can negatively impact real-time performance in autonomous driving applications. Therefore, with the current 1-way 4090 GPU resources, the InternImage encoder-based LSS at a 224×480 input resolution is the most reasonable choice for real-time operation. To select a larger input image size while maintaining real-time performance, further model optimization or more advanced GPU resources would be required. The HDMapNet model demonstrated the lowest latency performance compared to LSS and BEVFormer, suggesting it may be more suitable for real-time applications. However, despite its lower latency, HDMapNet recorded lower mIoU across all test sets compared to LSS and required more memory, which may limit its safety and feasibility for practical deployment in autonomous driving systems.

**Figure 8:**
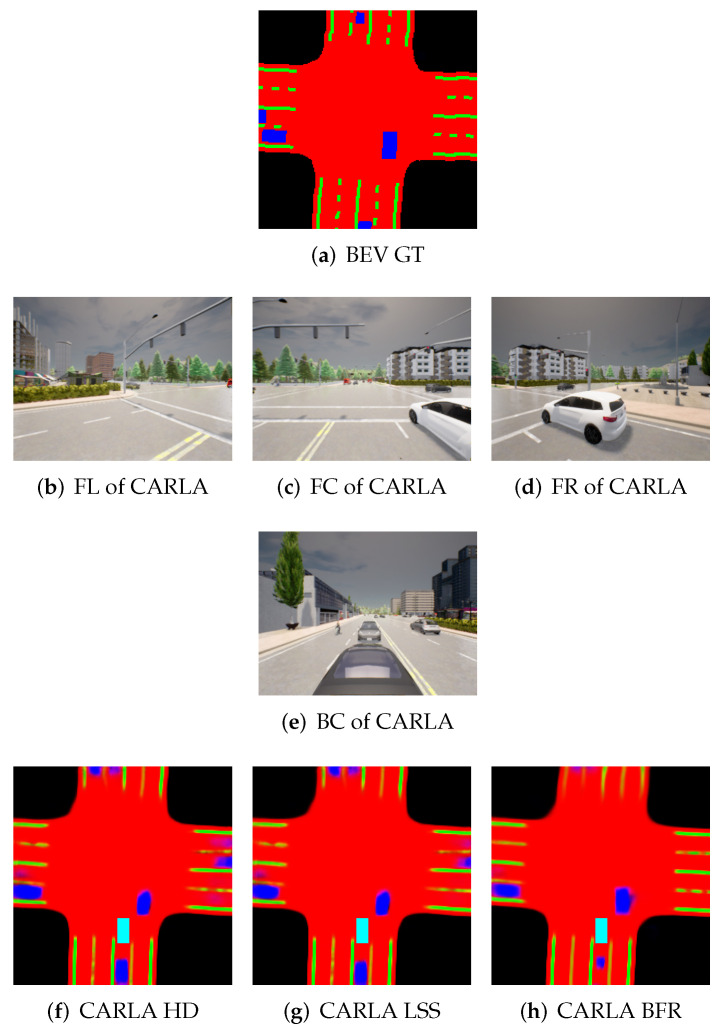
Examples of surround view images and corresponding BEV images using the segmentation model on the CARLA dataset (cyan: ego vehicle, red: drivable area, blue: car, and green: lane).

The aforementioned experiment was extended to a different environment, specifically the car learning to act (CARLA) simulator [26], where the scenario was configured using Town05 with an average speed of 14 km/h under high traffic conditions. Driving data were collected accordingly, and inference was performed using a BEV segmentation model pretrained on the nuScenes dataset, enabling cross-dataset performance validation. Table 6 presents the performance of BEV segmentation models across different input resolutions (224×480, 448×800, 672×1200). Figure 8 shows example outputs for the 224×480 resolution in the CARLA environment. While the nuScenes dataset provides real-world driving scenes, the CARLA simulator offers synthetic data with inherently different visual characteristics. Nevertheless, the overall performance trends observed across the two domains remain largely consistent. Notably, the CARLA environment, characterized by higher vehicle speeds, dynamic surroundings, and heavy traffic conditions, demonstrated improved performance at higher input resolutions (672×1200), particularly in recognizing small and densely distributed objects. In addition, it was observed that BEVFormer, which performs both temporal and spatial alignment during dynamic driving scenarios, achieves the best performance among the evaluated models.

Then, the performance was analyzed and improved from the perspective of data augmentation. Table 7 presents a performance comparison of data augmentation techniques and no data augmentation for the LSS model, with the ITN-T encoder and an input resolution of 448×800, which demonstrated the best performance efficiency. Various data augmentation techniques (e.g., cropping, resizing, and blurring) were applied, but the best performance was observed in the absence of data augmentation. This result suggests a correlation with the integrity of the original image acquisition. Specifically, it implies that minimizing noise introduced by the camera lens or internal circuitry may be critical for the effective operation of camera-based perception systems and related autonomous driving applications. In particular, applying data augmentation directly to the original PV images can lead to information distortion during the subsequent transformation to the BEV, thereby negatively impacting the final accuracy. More specifically, when projecting PV images onto the BEV space using camera intrinsics and extrinsics through geometric transformation matrices, the distortions introduced by PV image augmentations often act as noise in the final output. Moreover, if the augmentation is not applied consistently and temporally across all frames, it can adversely affect methods such as BEVFormer, which relies on temporal alignment. Therefore, rather than applying naive augmentations to PV images, it is more desirable to adopt geometric transformation-aware augmentation strategies, as suggested in [27].

### 5.3. Model Compression

Table 8 presents the results of applying FP16 quantization to the models in Table 2, while Table 9 shows the performance of these FP16-quantized models when deployed on an NVIDIA AGX Orin device under two power modes, 30 W and 50 W.

After applying FP16 quantization, the data type is reduced from FP32 to FP16, resulting in approximately a 50% reduction in model size and an average reduction of 1 ms in latency. Meanwhile, the mIoU performance of LSS, HDMapNet, and BEVFormer remains either the same or very similar. This suggests that the application of FP16 quantization is recommended as an optimization technique that ensures both performance and efficiency.

The on-device results in Table 9 show that, while the mIoU remains consistent with Table 8, the latency increases due to the power limitations. For example, with a 448×800 input resolution and quantized LSS using the ITN-T encoder, latency measurements were 51.9 ms on a single RTX 4090 GPU, 582.0 ms on the NVIDIA AGX Orin at 50 W, and 1167.3 ms at 30 W. Compared to the single RTX 4090 GPU, latency increased by 1021.4% at 50 W and 2149.1% at 30 W on the AGX Orin, which is insufficient for real-time performance in autonomous driving applications.

These results demonstrate that power supply plays a critical role in reducing latency. To ensure real-time decision-making in autonomous driving, sufficient power must be provided through high-capacity batteries. Furthermore, under limited power conditions, model compression and advancements in hardware performance are essential for minimizing latency. From a power efficiency perspective, while the single RTX 4090 GPU, requiring a minimum of 800 W, consumes 51.9ms×800W=41.5Ws, the NVIDIA AGX Orin consumes 582.0ms×50W=29.1Ws at 50 W and 1167.3ms×30W=35.0Ws at 30 W. This indicates that achieving low latency with high-performance GPUs consumes more energy, reducing energy efficiency. Conversely, deploying autonomous driving applications on embedded hardware improves energy efficiency but may lead to reduced latency performance.

Let us evaluate the performance with a 448×800 input resolution and ITN-T encoder. Although HDMapNet has the lowest mIoU performance, it shows an average latency reduction of 14.6% at 30 W and 21.9% at 50 W compared to LSS, indicating that HDMapNet is the most favorable option for real-time systems.

In summary, for autonomous driving systems that require more stringent real-time performance (latency below 30 ms), it is recommended to use the InternImage-T image encoder-based lift–splat–shoot technique with an input size of 224×480. On the other hand, for systems that demand higher accuracy performance (above 50 mIoU), the InternImage-T image encoder-based lift–splat–shoot technique with an input size of 448×800 is advised. Additionally, all these configurations require sufficient power supply to achieve optimal latency performance. Future research should focus on applying lightweight acceleration techniques to the InternImage-T image encoder and Voxel-based view transformation module to further minimize LSS latency.

## 6. Discussion

This section aims to discuss various special cases that were not covered in the previous experiments or require more in-depth analysis, along with potential solutions to address the associated challenges.

### 6.1. Failure Cases and Limitations: Heavy Traffic

An analysis of the failure cases in Figure 3 reveals that many occur under heavy traffic conditions. In such scenarios, the target objects tend to be small, closely spaced, and densely distributed, making accurate recognition particularly challenging. To maintain high segmentation accuracy under these conditions, increasing the input image resolution, as demonstrated in Figure 4 and Table 6, proves to be beneficial. Additionally, employing a more expressive backbone, such as InternImage, which can capture fine-grained object features more effectively, is also critical.

### 6.2. High-Speed Driving

High-speed autonomous driving environments can introduce several technical challenges to the perception accuracy of camera-based BEV segmentation. These issues primarily stem from the limitations of conventional image sensors in capturing rapidly changing scenes. In this section, we discuss the potential problems that may arise under high-speed conditions and propose possible solutions.

One of the most prominent issues in high-speed scenarios is motion blur [28]. Motion blur occurs when the camera or surrounding objects move rapidly while the shutter is open, resulting in smeared or distorted image features. This particularly affects the clarity of object boundaries. Since BEV transformation relies on the accurate spatial localization of features, motion blur can severely degrade the quality of the BEV map and reduce the accuracy of the segmentation output.

In addition, if the shutter speed is insufficient, frames may be distorted or fail to capture critical visual information. Cameras employing rolling shutters [29], which capture images line-by-line rather than all at once, are especially vulnerable to geometric distortions under fast motion. Such distortions compromise the consistency of object shapes and negatively affect both pixel-wise semantic segmentation and the accuracy of projection into the BEV plane.

Furthermore, high-speed driving increases the likelihood of temporal misalignment between frames due to significant object displacement across time. This poses a challenge to temporal fusion-based BEV segmentation methods such as BEVFormer, which rely on consistent temporal attention across frames. When object features are misaligned due to rapid motion or visual artifacts, temporal associations may become inaccurate, resulting in degraded performance in dynamic scenes.

Beyond these visual distortions, real-time constraints become more stringent in high-speed scenarios. As demonstrated in Fast-BEV [30], the trade-off between accuracy and latency becomes a critical concern. While real-time performance around 20–30 fps is essential for safe autonomous operation, maintaining this frame rate without sacrificing segmentation accuracy under high-speed motion remains a significant challenge.

Moreover, datasets such as nuScenes are primarily designed for realistic urban driving scenarios, typically involving speeds below 30 km/h. This results in a lack of diversity for high-speed conditions, representing a limitation in training and evaluating models under such scenarios. Therefore, the development of BEV segmentation datasets specifically tailored to high-speed driving environments will be a crucial challenge in future research.

Recent studies [31] have proposed event-based cameras as a promising alternative to mitigate motion blur. These sensors provide extremely high temporal resolution and low latency, enabling clear object boundaries even during rapid motion. However, event-based cameras have not yet been widely adopted in large-scale BEV segmentation tasks, and further research is needed to integrate them effectively into existing perception pipelines.

### 6.3. Inference Acceleration

The proposed BEV segmentation model demonstrates competitive accuracy across both real-world and simulated environments (nuScenes and CARLA). However, the inference speed presents limitations in fully meeting the real-time processing requirements of autonomous driving systems. This limitation becomes more critical in high-speed driving or complex traffic scenarios, where delayed perception and decision-making can directly impact driving safety.

One of the primary directions for improvement is the reduction of model complexity through knowledge distillation (KD). This technique enables a lightweight student model to learn the representational capacity of a high-performance teacher model, effectively reducing model size and computational load without significant degradation in performance. It has been shown that incorporating intermediate feature supervision further enhances KD performance for dense prediction tasks such as segmentation [22,23].

From a hardware perspective, utilizing modern high-performance GPUs (e.g., RTX 5080 and 5090) [32] can help strike a better balance between accuracy and latency. These platforms offer high computational throughput with low power consumption, and when supported by sufficient battery power, they are well-suited for real-time inference. Furthermore, designing dedicated neural processing units (NPUs) tailored to BEV segmentation could significantly accelerate specific operations, such as 2D-to-3D projection, multi-head attention, and high-resolution convolution [33,34].

At the system level, optimization of the middleware pipeline within the software-defined vehicle (SDV) architecture plays a critical role. Techniques such as pipeline scheduling optimization, asynchronous computation, and context-aware selective inference (e.g., triggering high-resolution inference only in complex scenes such as intersections or merging situations) can enhance overall system efficiency. Additionally, a hybrid inference strategy that dynamically adjusts input resolution based on driving speed and scene complexity can provide an effective trade-off between accuracy and real-time performance.

Ensuring real-time BEV segmentation requires a holistic optimization strategy across model design, hardware deployment, and system-level integration. Future work will focus on developing an end-to-end optimized framework that combines these approaches to meet the demands of practical autonomous driving systems.

### 6.4. Sensor Innovation

In addition to algorithmic improvements, achieving accurate and reliable BEV perception in real-world autonomous driving, particularly in camera-based high-speed and dynamic environments, requires innovation at the sensor level. Conventional RGB cameras face inherent limitations in temporal resolution and sensitivity to fast motion, which can lead to degraded performance in BEV transformation and segmentation. This section outlines key sensor-level improvements to overcome these challenges.

One of the most direct and effective approaches is to increase the frame rate of image sensors. Higher frame rates help reduce motion blur and alleviate temporal misalignment between frames. By capturing finer-grained object movements, they enable more accurate recognition of fast-moving object boundaries. While standard automotive cameras typically operate at 30–60 fps, increasing this to 90–120 fps can significantly enhance perception under high-speed driving conditions. However, higher frame rates inevitably result in greater data throughput and processing latency. Therefore, FPGA-based or dedicated hardware accelerators for preprocessing should be employed in parallel.

Another promising approach is the use of temporal offset compensation in multi-camera systems. Traditional multi-camera setups typically employ synchronized image capture across all cameras. However, under high-speed motion, such synchronization may lead to redundant information with limited temporal diversity. To address this, intentional millisecond-scale time offsets (Δt) can be introduced between cameras. For instance, front-left and front-right cameras may be configured to capture images 5–10 ms apart. Fusing these temporally staggered frames effectively increases the system’s temporal resolution and enriches the temporal representation of the scene. Alignment and fusion strategies that account for these offsets can significantly contribute to generating more temporally aware BEV representations.

A third direction is the use of event-based cameras, as previously discussed. Unlike traditional frame-based cameras, event cameras operate asynchronously by recording pixel-level changes in brightness, only when such changes occur. These sensors provide microsecond-level temporal resolution and extremely low latency, enabling sharp perception of object boundaries without motion blur, even during high-speed motion or in low-light conditions. While the output of event cameras is highly sparse, recent advances in event-based convolutional neural networks (CNNs) and spiking neural networks have demonstrated promising results for processing such data [35].

Lastly, to compensate for the limitations of cameras in high-speed scenarios, sensor fusion with inertial measurement units (IMUs) can be highly beneficial. IMUs provide real-time ego-motion estimates, which can be fused with visual data to correct motion-induced image distortions, thereby improving the stability and accuracy of BEV perception.

## 7. Conclusions

This paper conducted a mathematical analysis and comparative evaluation of the three major approaches to BEV segmentation technology for autonomous driving: depth-based methods, MLP-based methods, and transformer-based methods, focusing on their representative techniques, e.g., lift–splat–shoot, HDMapNet, and BEVFormer. The performance of these methods was evaluated using the nuScenes dataset, and optimization was performed across three stages in terms of accuracy, latency, and model size.

In the first stage, encoder, view transformation, and decoder models were selected to maximize mIoU performance. In the second stage, environmental optimization was performed through input resolution adaptation and data augmentation. In the third stage, model compression was applied to ensure efficient deployment on embedded systems. Experimental results from the first stage demonstrated that the lift–splat–shoot VT model with the InternImage encoder and EfficientNet-B0 decoder achieved the highest mIoU performance with an input image size of 448×800. Notably, the lift–splat–shoot VT model with the InternImage-T encoder and EfficientNet-B0 decoder provided high mIoU performance with excellent efficiency. The application of the second stage revealed that 448×800 is the optimal input resolution, showing that the best resolution depends on the model and dataset characteristics. On the other hand, latency remained relatively constant as input resolution increased. To address the challenges associated with the crosswalk and car classes, which emerged as bottlenecks for overall performance, it was determined that acquiring pristine, noise-free images and utilizing sophisticated models like InternImage is advantageous. Additionally, it was found that data augmentation had minimal effect on performance improvement.

In the third stage, model compression using FP16 quantization resulted in a 50% reduction in memory size and decreased latency, while maintaining similar or identical mIoU performance. When deployed on an NVIDIA AGX Orin device with power limitations, the model showed improved energy efficiency, though latency increased depending on the power supply. To overcome this, further research is needed on providing sufficient battery power, lightweight acceleration techniques for the BEV segmentation model, and hardware acceleration.

In conclusion, for autonomous driving systems that require more stringent real-time performance (latency below 30 ms), it is recommended to use the InternImage-T image encoder-based lift–splat–shoot technique with an input size of 224×480. Conversely, for systems that demand higher accuracy performance (above 50 mIoU), the InternImage-T image encoder-based lift–splat–shoot technique with an input size of 448×800 is recommended. Compared to the original lift–splat–shoot technique, this approach achieves a 29.2% higher mIoU, while maintaining similar latency performance and reducing memory size by 32.2%.

## Figures and Tables

**Table 1:** Performance comparison of ResNet-50 with various quantization techniques on the CIFAR-10 dataset.

Quantization Model	Acc (%)	Size (KB)	Lat (ms)
NQ	80.7	94,052	177
BLQ	80.3	24,161	2348
FIQ	80.4	24,269	2078
F16	80.6	47,072	152
QAT	80.5	24,281	2069

**Table 2:** The mIoU, latency, and size performance according to image encoder model (latency: ms; size: MB).

Model	LSS (224 × 480)					
	EFN-B0	EFN-B4	MiT-B0	MiT-B2	ITN-T	ITN-B
mIoU	46.4	47.7	44.8	46.2	51.3	52.2
Latency	21.9	30.5	19.7	25.8	30.6	37.7
Size	59.4	116.1	51.6	138.5	159.2	427.0
Lane	53.2	53.9	51.8	53.1	58.8	59.7
Crosswalk	42.3	44.1	39.7	40.7	51.5	52.1
Bound	57.2	58.6	55.6	56.8	63.0	63.9
Car	32.9	34.3	32.2	34.3	37.9	39.2
Model	HMN (224 × 480)					
	EFN-B0	EFN-B4	MiT-B0	MiT-B2	ITN-T	ITN-B
mIoU	42.3	43.7	39.9	38.7	47.1	48.6
Latency	22.4	30.9	20.0	25.9	30.4	33.6
Size	117.6	171.6	110.0	190.6	212.3	471.2
Lane	50.4	51.2	47.6	45.9	54.4	55.4
Crosswalk	37.3	39.4	35.6	33.2	44.0	46.0
Bound	53.5	55.0	50.7	48.9	58.1	59.8
Car	28.0	29.4	25.9	26.9	31.9	33.3
Model	BFR (224 × 480)					
	EFN-B0	EFN-B4	MiT-B0	MiT-B2	ITN-T	ITN-B
mIoU	35.3	40.1	42.3	39.3	48.0	50.2
Latency	39.1	49.1	34.0	39.2	47.5	53.5
Size	66.5	76.4	76.12	157.5	179.15	439.3
Lane	41.5	46.3	49.1	46.7	54.6	56.7
Crosswalk	29.9	34.5	37.6	31.5	44.6	47.2
Bound	45.2	50.3	53.0	51.0	58.4	60.8
Car	24.9	29.5	29.8	28.4	34.5	36.2
Model	LSS (448 × 800)					
	EFN-B0	EFN-B4	MiT-B0	MiT-B2	ITN-T	ITN-B
mIoU	49.1	48.3	47.7	48.3	53.1	54.9
Latency	30.1	50.1	28.8	50.0	51.7	95.5
Size	59.7	116.4	51.9	138.8	159.5	427.3
Lane	54.6	55.3	54.5	55.8	58.8	60.5
Crosswalk	46.1	45.7	43.2	45.0	51.0	53.6
Bound	59.6	60.0	58.1	59.6	62.8	64.5
Car	36.1	38.3	35.0	33.1	40.0	41.2
Model	HMN (448 × 800)					
	EFN-B0	EFN-B4	MiT-B0	MiT-B2	ITN-T	ITN-B
mIoU	44.1	43.2	42.5	43.2	45.8	50.7
Latency	25.5	39.1	23.1	38.9	40.2	83.7
Size	153.5	207.5	145.7	226.5	248.2	507.1
Lane	51.4	50.2	49.7	49.4	52.2	57.3
Crosswalk	40.2	38.2	37.3	38.6	42.1	48.1
Bound	54.8	53.9	29.6	54.2	56.1	60.8
Car	30.2	30.5	42.5	30.8	33.0	36.6
Model	BFR (448 × 800)					
	EFN-B0	EFN-B4	MiT-B0	MiT-B2	ITN-T	ITN-B
mIoU	38.9	26.7	44.1	44.8	50.8	53.9
Latency	43.3	60.4	44.3	65.7	64.5	106.8
Size	66.5	76.4	76.12	157.5	179.14	439.3
Lane	44.3	31.2	50.7	51.7	56.8	59.8
Crosswalk	34.1	18.5	39.6	39.9	47.7	51.9
Bound	49.1	35.9	54.2	55.5	60.5	63.7
Car	28.3	21.2	31.9	32.0	38.2	40.1

**Table 3:** Performance of mIoU, latency (ms), and size (MB) for various decoder models in the LSS ITN-T encoder model.

Model	EFN-B0	MiT-B0	ITN-T
mIoU	51.3	50.7	51.9
Latency	30.6	28.5	34.2
Size	159.2	151.8	254.0
Model	EFN-B4	MiT-B4	ITN-B
mIoU	52.0	51.1	52.7
Latency	32.1	33.5	41.2
Size	213.9	232.3	512.9

**Table 4:** ImageNet top-1 performance per encoder.

Encoder Model	Top-1 Acc (%)	Params (M)
EFN-B0	77.1	5.3
EFN-B4	82.9	19.0
MiT-B0	70.5	3.7
MiT-B2	81.6	25.4
ITN-T	83.5	30.0
ITN-B	84.9	97.0

**Table 5:** Performance of BEV segmentation models using the ITN-T image encoder with 672×1200 input image size in terms of mIoU, latency, and size (latency: ms; size: MB).

Model	LSS	HMN	BFR
mIoU	52.7	48.1	52.8
Latency	197.2	68.2	209.5
Size	160.3	312.4	179.1

**Table 6:** Comparison of BEV segmentation performance using the ITN-T image encoder on the CARLA dataset.

224 × 480	HMN	LSS	BFR
mIoU	62.1	64.0	65.5
DAS	89.2	89.1	89.1
Lane	44.1	47.1	49.1
Vehicle	53.2	56.0	58.4
448 × 800	HMN	LSS	BFR
mIoU	62.8	65.5	67.5
DAS	89.4	91.0	91.7
Lane	45.1	48.1	51.7
Vehicle	54.0	57.4	59.1
672 × 1200	HMN	LSS	BFR
mIoU	64.3	65.6	67.9
DAS	90.5	91.7	91.1
Lane	46.0	48.1	52.0
Vehicle	56.4	57.1	60.7

**Table 7:** The performance of LSS ITN-T BEV segmentation with data augmentation on a 448×800 input image size (latency: ms; size: MB).

Model	LSS DataAug	HMN DataAug	BFR DataAug
mIoU	51.6	43.2	49.3
Latency	51.3	40.6	64.2
Size	160.3	312.4	179.1

**Table 8:** Optimization performance through FP16 quantization (latency: ms; size: MB).

Model	Quantized LSS (224 × 480)					
	EFN-B0	EFN-B4	MiT-B0	MiT-B2	ITN-T	ITN-B
mIoU	44.7	45.9	43.1	44.2	49.0	49.9
Latency	21.7	30.2	19.4	25.6	30.2	37.4
Size	29.7	58.0	25.8	69.2	79.6	213.5
Model	Quantized HDMapNet (224 × 480)					
	EFN-B0	EFN-B4	MiT-B0	MiT-B2	ITN-T	ITN-B
mIoU	42.3	43.7	39.8	38.7	47.1	48.6
Latency	22.2	30.7	19.9	25.7	30.2	33.4
Size	58.8	85.8	55.0	95.3	106.2	235.6
Model	Quantized BEVFormer (224 × 480)					
	EFN-B0	EFN-B4	MiT-B0	MiT-B2	ITN-T	ITN-B
mIoU	33.8	38.6	40.8	37.8	46.5	48.7
Latency	39.0	48.9	33.9	39.0	47.3	53.3
Size	33.2	38.2	38.1	78.8	89.6	219.6
Model	Quantized LSS (448 × 800)					
	EFN-B0	EFN-B4	MiT-B0	MiT-B2	ITN-T	ITN-B
mIoU	47.1	46.1	45.8	46.4	51.3	53.0
Latency	29.8	49.8	28.6	49.8	51.9	95.3
Size	29.8	58.2	26.0	69.4	79.8	213.6
Model	Quantized HDMapNet (448 × 800)					
	EFN-B0	EFN-B4	MiT-B0	MiT-B2	ITN-T	ITN-B
mIoU	44.1	43.2	42.5	43.1	45.8	50.7
Latency	25.7	38.9	22.8	38.8	40.1	83.6
Size	76.8	103.8	72.8	113.2	124.1	253.6
Model	Quantized BEVFormer (448 × 800)					
	EFN-B0	EFN-B4	MiT-B0	MiT-B2	ITN-T	ITN-B
mIoU	37.4	25.2	42.5	43.3	49.3	52.4
Latency	43.2	60.0	44.1	65.5	64.3	106.7
Size	33.2	38.2	38.1	78.8	89.6	219.6

**Table 9:** On-device performance according to power limitation (latency: ms).

Model	Quantized LSS (224 × 480)					
	EFN-B0	EFN-B4	MiT-B0	MiT-B2	ITN-T	ITN-B
mIoU	44.7	45.9	43.1	44.2	49.0	49.9
Latency (30 W)	182.8	321.1	170.5	289.7	392.6	634.2
Latency (50 W)	136.4	204.9	124.8	177.9	216.9	319.7
Model	Quantized HDMapNet (224 × 480)					
	EFN-B0	EFN-B4	MiT-B0	MiT-B2	ITN-T	ITN-B
mIoU	42.3	43.7	39.8	38.7	47.1	48.6
Latency (30 W)	141.9	283.1	136.6	249.5	353.5	587.1
Latency (50 W)	124.4	157.8	112.8	132.7	153.1	255.4
Model	Quantized BEVFormer (224 × 480)					
	EFN-B0	EFN-B4	MiT-B0	MiT-B2	ITN-T	ITN-B
mIoU	33.8	38.6	40.8	37.8	46.5	48.7
Latency (30 W)	820.0	946.8	825.9	917.9	964.0	1283.6
Latency (50 W)	393.3	399.7	299.3	413.9	432.8	600.9
Model	Quantized LSS (448 × 800)					
	EFN-B0	EFN-B4	MiT-B0	MiT-B2	ITN-T	ITN-B
mIoU	47.1	46.1	45.8	46.4	51.3	53.0
Latency (30 W)	448.1	929.7	494.6	886.7	1167.3	1931.4
Latency (50 W)	324.1	537.4	311.6	489.8	582.0	901.1
Model	Quantized HDMapNet (448 × 800)					
	EFN-B0	EFN-B4	MiT-B0	MiT-B2	ITN-T	ITN-B
mIoU	44.1	43.2	42.5	43.1	45.8	50.7
Latency (30 W)	319.4	756.8	329.8	706.3	997.2	1743.4
Latency (50 W)	211.5	404.9	200.2	346.9	454.4	732.4
Model	Quantized BEVFormer (448 × 800)					
	EFN-B0	EFN-B4	MiT-B0	MiT-B2	ITN-T	ITN-B
mIoU	37.4	25.2	42.5	43.3	49.3	52.4
Latency (30 W)	980.7	1128.1	930.6	1353.4	1669.9	2311.7
Latency (50 W)	457.0	618.2	464.2	615.26	714.4	1059.4

## Data Availability

The original contributions presented in this study are included in the article. Further inquiries can be directed to the corresponding author.
